# Perspectives on key principles of generalist medical practice in public service in sub-saharan africa: a qualitative study

**DOI:** 10.1186/1471-2296-12-67

**Published:** 2011-07-04

**Authors:** Stephen J Reid, Robert Mash, Raymond V Downing, Shabir Moosa

**Affiliations:** 1 Primary Health Care Directorate, University of Cape Town, Cape Town, South Africa; 2 Division of Family Medicine and Primary Care, University of Stellenbosch, Tygerberg, South Africa; 3 Department of Family Medicine, Moi University, Eldoret, Kenya; 4 Johannesburg Metro Health Services, Johannesburg, South Africa

## Abstract

**Background:**

The principles and practice of Family Medicine that arose in developed Western countries have been imported and adopted in African countries without adequate consideration of their relevance and appropriateness to the African context. In this study we attempted to elicit *a priori *principles of generalist medical practice from the experience of long-serving medical officers in a variety of African counties, through which we explored emergent principles of Family Medicine in our own context.

**Methods:**

A descriptive study design was utilized, using qualitative methods. 16 respondents who were clinically active medical practitioners, working as generalists in the public services or non-profit sector for at least 5 years, and who had had no previous formal training or involvement in academic Family Medicine, were purposively selected in 8 different countries in southern, western and east Africa, and interviewed.

**Results:**

The respondents highlighted a number of key issues with respect to the external environment within which they work, their collective roles, activities and behaviours, as well as the personal values and beliefs that motivate their behaviour. The context is characterized by resource constraints, high workload, traditional health beliefs, and the difficulty of referring patients to the next level of care. Generalist clinicians in sub-Saharan Africa need to be competent across a wide range of clinical disciplines and procedural skills at the level of the district hospital and clinic, in both chronic and emergency care. They need to understand the patient's perspective and context, empowering the patient and building an effective doctor-patient relationship. They are also managers, focused on coordinating and improving the quality of clinical care through teamwork, training and mentoring other health workers in the generalist setting, while being life-long learners themselves. However, their role in the community, was found to be more aspirational than real.

**Conclusions:**

The study derived a set of principles for the practice of generalist doctors in sub-Saharan Africa based on the reported activities and approaches of the respondents. Patient-centred care using a biopsychosocial approach remains as a common core principle despite wide variations in context. Procedural and hospital care demands a higher level of skills particularly in rural areas, and a community orientation is desirable, but not widely practiced. The results have implications for the postgraduate training of family physicians in sub-Saharan Africa, and highlight questions regarding the realization of community-orientated primary care.

## Background

Family Medicine is in the process of developing rapidly as an academic discipline in Africa. Academic departments of Family Medicine are being established in many countries, and undergraduate and postgraduate training programmes are being offered, with the aim of producing competent generalist doctors who can function within and improve the quality of primary care. But what model or conceptual framework underlies these initiatives? Is it appropriate for Africa, or are we unquestioningly importing a foreign notion of what Family Medicine should be in our context? This project set out to establish *a priori *principles on which the discipline could be founded in an African setting.

The World Health Report for 2008 *Primary Health Care: Now More Than Ever *[1] emphasises the importance of service delivery reform. Conventional outpatient care has focused on episodic curative care while disease control programmes have focused on specific priority diseases. The report argues for a more people-centred primary care that focuses on health needs in a more holistic way and within an enduring personal relationship. Care should be comprehensive, continuous and person-centred with responsibility of the primary care team for the health of the community served and not just the patient in front of them. Family Medicine has a long tradition of promoting these principles and training doctors in this more person-centred and community-orientated approach [2].

This modern Family Medicine had its origins in the countries of Europe and North America, and was developed in response to the specific health care needs arising in those countries in the second half of the 20^th ^Century [3]. In Sub-Saharan Africa however, the health care needs are different from those that Europe and North America faced only a generation ago when they developed the discipline [4]. Firstly there is a high burden of infectious disease, nutritional deficiency and trauma as well as maternal and childhood illness [5]. Secondly, African healthcare systems have fewer resources and are organised differently to other parts of the world, and the role of the family physician may also be different. Not surprisingly, as Family Medicine is now spreading rapidly in Sub-Saharan Africa [6], many scholars have raised questions about how closely African Family Medicine should or will follow the models developed in the north,[7,8] and a report of a new Family Medicine training programme in Africa described it as having 'few similarities to North American family medicine education'[9].

Furthermore, many countries in Sub-Saharan Africa rely on district hospitals run by generalists to bring hospital care close to the community. First contact care in the public service is usually provided by a nurse or mid-level worker and not a doctor. The family physician's role therefore will differ from that in European or American contexts, where most of the current family medicine literature is generated. In Sub-Saharan Africa family physicians and generalist medical officers are likely to need more surgical, anaesthetic and procedural skills to provide services at the district hospital [10], as well as skills in consulting, mentoring and teaching to support the front line primary care workers.

There is therefore a need for the discipline of family medicine in an African context to reach consensus on a regional definition that can articulate what family medicine has to offer to communities, local governments and health systems. One such consensus definition was developed at the regional WONCA (World Organization of National Colleges and Academies of Family Medicine) conference in October 2009 [11]. Part of the process of developing this definition was a Delphi study with teachers of family medicine and graduates of family medicine programmes throughout sub-Saharan Africa [12]. This study quantified the consensus of this expert group on the principles of family medicine. The panel identified 50 relevant principles and ranked them not only in order of importance, but also in terms of whether they are currently seen in-action or are still largely theoretical.

The Delphi study, however, framed the question using the existing world literature on family medicine and obtained consensus from a panel which represented a more academic view and which might have simply re-iterated existing ideas from the north. By contrast, in this study we explore a more grassroots and experientially-based viewpoint on the role of the generalist doctor in sub-Saharan Africa. This perspective of established and experienced generalist doctors who have not had formal training in Family Medicine, on their role in their health systems can be triangulated with the results of the previous study to give a more complete and textured picture of the principles of family medicine in the Africa context.

The decision to study the roles and principles of generalist practice as one way to contribute to the development of Family Medicine in Africa is an overt departure from previous studies. There is an assumption in the literature, often unstated, that Family Medicine world-wide is the same as Primary Care [13], and that characteristics such as first contact care and longitudinal ongoing care will be core to Family Medicine anywhere [14]. While we agree that 'a strong primary health care system is essential to provide effective and efficient health care in both resource-rich and in resource-poor countries' [15], the role that Family Medicine should play in that system cannot be assumed to be the same in Africa as in developed countries [4].

One approach to health care needs in Africa is to assume northern standards: 'An analysis of standards for the best practice of family medicine in Northern European countries provides a framework for identifying the difficulties and deficiencies in the health services of developing countries, and offers strategies and criteria for improving primary health care practice'[16]. While that may have value, our assumption is that the experience of long-serving medical officers in a variety of African counties can also provide *a priori *principles by which we can explore the development of the new discipline of Family Medicine in our own context.

This project was based on a network of established and developing academic departments of Family Medicine in sub-Saharan Africa, which had already been created. This network has been formed over the past 9 years with several VLIR Interuniversity Collaboration projects financed by the Belgian government through ICHO and the Primafamed Project [17] financed by the European Union through Edulink [18], with the aim of establishing an institutional network between departments of family medicine and primary health care in universities in Sub-Saharan Africa. The project is coordinated from Ghent University, Belgium and supports African universities in their development of training in family medicine. It embraces the principle of south-south cooperation, encouraging the sharing of unique knowledge and experience between African institutions. The network includes departments of family medicine of associated universities in South Africa, Ghana, Sudan, Nigeria, Kenya, Uganda, Tanzania, Democratic Republic of Congo, Rwanda, Swaziland, Lesotho, Botswana, Mozambique, Zambia, Malawi, and Namibia.

## Methods

The aim of the study was to explore the scope of practice, perceived roles and emergent principles of generalist medical practice in Africa. The study was driven by the research question 'What are the key principles by which experienced generalist doctors practice in the context of the public health service in Africa?'. An inductive qualitative study design was utilized, based on a series of open-ended interviews with experienced generalist doctors in different Sub-Saharan African countries who had never been exposed to formal training or teaching in family medicine or general practice.

Two potential respondents who met the inclusion criteria, were likely to offer relevant information and were willing to participate, were purposively identified by the Primafamed coordinator in each of the 16 countries supported by the Primafamed network. Respondents were generalist clinicians, usually termed Medical Officers, working across all medical disciplines and involved in patient care, either part-time or fulltime, over the previous 5 years. They had to have worked at least 5 years in the public service or in a non-profit organization. Excluded from the study were fulltime private practitioners who did not work in the public sector at all, and District Medical Officers who did no clinical work, as well as any medical officers who had completed post-graduate specialization, including general practice or family medicine training.

Written informed consent was obtained from all respondents before the interviews, which lasted 30 to 60 minutes. Basic demographic information was recorded initially, as well as other data such as position, length of time in practice, and qualifications. Open-ended, exploratory, in-depth individual interviews were then conducted by the interviewers one-on-one, and recorded digitally. All the interviews were conducted in English according to the preference of the respondents, with the exception of one in Rwanda which was conducted in Kinyarwanda and translated to English by the interviewer. All digital recordings were transcribed verbatim by the interviewers. Interviewers were identified with the help of the Primafamed co-ordinators in each country, and the researchers themselves also acted as interviewers. The investigators conducted two 2-day training sessions in qualitative interviewing, one in Nairobi, Kenya and the other in Johannesburg, South Africa. A standard operating procedure guided the whole process, and a standard interview guide was used for prompting during the interviews.

The following open-ended questions were used to explore the scope of practice, perceived roles and emergent principles of generalist medical practice.

• Please describe to me the most significant features of the health system in your country.

• Can you tell me how you typically spend your week? Please describe your major activities starting with the one you spend most time on.

• How would you describe your role as a generalist doctor?

• What is important to you in your work?

• Please explain the most important principles for you in your approach to your work

• What changes in your scope of practice and your role would make you more effective in the district?

Analysis of the data was done using the framework approach [19]. Firstly an initial reading of the transcripts allowed each researcher to familiarize themselves with the data, and to identify major and minor themes independently. Secondly at a meeting of the research team these themes were then prioritized and debated, and a thematic index developed. The index was structured into three main sections:

• External collective: Themes relating to the broader societal and organisational context.

• Internal individual: Individual values and beliefs directly expressed by the respondents

• External individual: Individual roles, activities and behaviours described by the respondents and from which one might interpret underlying individual values and beliefs.

All the authors then systematically coded the text from each transcript according to the index. Three charts were then created based on the three sections described above and data with the same code from all the transcripts charted in the same column. Data was either quoted directly or the main point summarised with reference to the source. The research team then met together to interpret and analyze the data collated on the charts.

The protocol was approved by the University of KwaZulu-Natal Biomedical Research Ethics Committee (ref BE017/09). Respondents were not offered any monetary reward for participating in the study. The data produced in the project remains confidential, and respondents remain anonymous in all transcripts and analyses. Subjects and countries were assigned codes, and care was taken not to identify the data by country in order to protect confidentiality.

## Results

A total of 16 interviews were conducted by 6 different interviewers in the following countries: South Africa, Lesotho, Botswana, Swaziland, Kenya, Uganda, Rwanda and Ghana. After 16 interviews were completed it was decided by the research team that there was sufficient data for analysis. Suitable respondents in the other countries in the Primafamed network were not able to be identified and interviewed within the time period allocated for data collection.

### Contextual issues: External Collective theme

All respondents were very aware of the constraints of their contexts and readily volunteered information on this theme. A common and often repeated statement concerned the general lack of resources to cope with the burden of illness in Africa. This related to the shortage of qualified staff, and lack of equipment as well as medication, and the resultant work overload of the few who do fill the posts. Under the best circumstances it leads to an innovative and adaptable approach of making the most out of what few resources there are. But most of the time it just leads to frustration and burnout, and contributes to the rapid turnover of staff.

*'We are short staffed on the nurses' side and with the doctors... we don't have enough time with each patient because of the pressure of have to push the queue'*.

'You have 40 to 60 patients to see per day'

The rural context of many of the respondents had both negative and positive aspects-although there were many frustrations, some respondents found satisfaction from working in under-served areas:

*'working in a small rural place I get a lot of satisfaction with working with rural people who need medical attention'*.

Traditional health beliefs formed part of the context. There was recognition that many patients used traditional healing before consulting the generalist, although attitudes varied from openness and acceptance to rejection and hostility.

*'Most of them resort to traditional treatment. Some consult the churches for prayer; most people believe that every disease in Africa is given by a close relative so they believe in the spiritualist first. Many of them come in critical condition'*.

In addition to resource constraints, there were also health system failures that created significant challenges:

*'When mistakes occur, it is a system failure not the level of competency'*.

Referral of patients to higher levels of care was a huge challenge, and required practitioners to constantly upgrade their skills, as they were often forced to deal with clinical problems beyond their comfort zones.

*'One is forced to extend one's capacity continuously'*.

Nevertheless, most asserted that generalists manage 90% of patients seen and only refer a minority.

With regard to career choice, it was interesting to note that most respondents were generalists by default and not by choice and would have preferred to specialize if they had had the opportunity.

*'It's not a choice for me to be a generalist; I would have liked to do something more than being a generalist'*.

The absence of a career path for a generalist clinician, and the lack of benefits in addition to the heavy workload, made this an unattractive career choice for clinicians in Africa.

### Internal Individual theme

The internal individual theme related to personal issues of motivation and principles, by which practitioners order their lives and do their work. Four sub-themes arose from the data in the interviews: motivation, attributes, national responsibility, and continuing professional development.

Motivation as a generalist was easily and well described. A clearly expressed drive was to make a difference, despite the context. It was felt important that they be *'making a difference to individual lives'*, and one described his work as *'a passion for assisting people'*.

Individual sources of motivation were described in different ways, such as:

*'from childhood I wanted to be a doctor. For me it was really important to help people. I can see it as I'm helping people'*.

'I'm a Christian guy so religion plays a role there'

*'You operate *[on] *a patient with a serious problem, and then you see him walking. You feel happy.'*

Respondents described the various attributes required of them as generalists, mostly regarding relationships with patients and colleagues, such as commitment, respect, empathy, caring, compassion, integrity and trust, but also other qualities:

*'I try to treat everybody with respect... it's about being a people's person'*.

'It's flexibility: it's the ability to multitask and improvise and to provide leadership.'

'You should be working as a warrior,...get tough though there are challenges, but we should accept......'

The important attributes were also described in terms of technical and organizational issues, such as thoroughness, knowing one's limits, having a systematic approach, being flexible and able to improvise and innovate.

*'You have to learn to listen to the patient and to take proper history from the patient... physical examination... investigation... differential diagnosis... actual diagnosis'*.

'You work as an individual but I also realized that you need some structure and assistance to get things done.'

There was also a sense of social and national responsibility that was expressed explicitly by some:

*'I feel that I have a role to play towards the development of the country, the well being of the people, this is why I still remain in government...'*.

*'I need to be part of a group of people that are responsible for finding solutions to the good health of all Rwandans'*.

There were also important personal connections to their context:

*'Then the environment, this is where I got married. So all these people I treated them as my what, my relatives*'.

'I grew up in a rural setting and because of that I know life in the rural areas can be meaningful. I feel I should have a contribution to the locals..'

One respondent proposed rewards and incentives for staff, including verbal positive feedback, certificates, trophies and relationships with the system.

'I need to be motivated externally by my bosses in the province. But internally mine is actually my work.'

Some were motivated by the challenges themselves:

*'But what motivates me especially in those rural facilities is the challenge of delivering the health services where access is a problem'*.

Ongoing learning was seen as driven by need, and this was seen to attract young doctors:

*'Continuous training is essential'*, and *'if we do not train, if we do not get more skills then you cannot be able to give the required quality of care to our patients'*.

Professional development was seen as self-directed: *'its all up to you'*. Learning was usually in the mode of supervised practice with colleagues, but there were also formal courses and lectures. Experienced medical officers requested recognition of their experience.

### External Individual theme

This theme relates to the visible roles, activities and behaviours of the respondents as generalist clinicians in public service in African countries. Relationships with their patients were seen as paramount, and they therefore placed high importance on relational skills. They were also quite aware of the social, economic, psychological and cultural issues that impact on a patient's illness:

'Most of our health problems are lifestyle diseases anyway. So if it's a lifestyle disease then these are socio-cultural issues.'

'Some patients they bring such pains because, well they are not actually bringing the pain but they are bringing the social burden they have in their lives and everything.'

'I do not manage diseases, I manage the patient... Sometimes... the problem the patient does not tell you... you listen and know the problems behind it.'

This understanding of the complex determinants of illness led to an appreciation of the need to listen carefully to patients:

'The principle is to get the history from the patient...in an open way so that the patient feels free...the patient must not feel that he has to tell you what you want to hear.'

Even beyond this, some spoke of the need for empathy with patients:

'you must stand in the boots of that person and you must, you must tailor what you do to that person according to that.'

Other equally important relational issues were mentioned: the need for trust, respect, and honesty with patients, the importance of confidentiality, and the value of a continuing relationship, especially with those with chronic diseases.

Respondents accepted the need to do hospital ward work, and even major surgical procedures. The lists of procedures varied from place to place. For some it was smaller out-patient procedures:

'circumcisions... minor surgery: small cases... small abscesses, cysts, incisions'

While for others it involved major surgery:

'appendicectomy, prostatectomy, laparotomy... c/sections, and oophorectomy.'

Another role that most respondents found themselves involved with is administration and management. Sometimes it was simply organizing and attending meetings for purposes such as continuous professional development, audits, or mortality reviews. Often the tasks went beyond this, and involved conflict management among staff members, problem solving of clinical issues, and *'thinking strategically' *to *'bridge the gap between planning at the district level and translation into reality at the coalface'*.

Some were in more defined leadership roles:

'It's like a sort of puppet master: you pull strings and make sure that everybody is in the right place at the right time.'

Not surprisingly, these additional management duties can lead to tension:

'The balance between clinical work and managerial work is difficult.'

Most respondents were also involved with teaching, especially for medical students, interns, and junior doctors, but also for nurses and community health workers. Most of this was done on the job, by mentoring, role-modeling, and clinical teaching, although a few gave lectures as well.

'Students .... participate, they become quite functional. So they just join the team and ..... the training they get is not that formal.'

'So I see my role now basically at the present moment is to, to help those, to teach them...to help themselves so after two, three, four months they will be sort of independent '

These were tasks that were accepted without the tension noted with management. Yet there was also a recognition of the need for these generalist 'teachers' to themselves be taught beyond medical school, either *'by extending the duration of the medical school even to cover 8 to 10 years'*, or *'post-medical school one year in every discipline...'*

Involvement in community health activities, however, revealed a different pattern. Most generalists affirmed in theory that community health was important and a few had been involved with outreach clinics or education to community groups. However, few, if any, of our respondents were actively involved with community health:

'there should be that link between the health workers and the community'

'We sit here, we don't go to the community, we only sit down and see patients as they come here.'

Finally, many respondents affirmed the importance of teamwork, both for hospital management and for district health management.

'What's important is to build a very good teamwork around your department, make sure every work goes on with minimal supervision.'

Some generalists seemed successful in encouraging and shepherding effective teams at their places of work. However, many admitted that there were major barriers to developing effective teams.

*'The health care system... would be excellent, but the problem is that apparently there is no team work...'*.

A particularly poignant admission was the difficulty doctors and nurses sometimes have working together. Nurses may resent doing things *'for the doctor'*, and one doctor felt:

'it's like you are asking them a favour to look after a patient.'

This could lead to anger toward nurses, which one doctor admitted was destructive of teamwork.

## Discussion

### Key findings of the study

A key principle can be defined as 'a belief that is accepted as a reason for acting or thinking in a particular way' [20]. A number of key principles can be derived from the findings. Generalist doctors in sub-Saharan Africa firstly need to cope with a context that is characterized by traditional health beliefs, resource constraints, high workload and the difficulty of referring patients to the next level of care. Secondly they need to be competent across a wide range of clinical disciplines and procedural skills at the level of the district hospital and clinic, in both chronic and emergency care. They need to demonstrate relational and communication skills in terms of understanding the patient's perspective and context, empowering the patient and building an effective doctor-patient relationship, as well as in forming, participating in and leading teams of health workers. They also need to be effective managers, focusing on co-ordinating, maintaining and improving the quality of clinical care through teamwork. They need to perform well as clinical trainers and mentors of other health workers who are competent to share in the workload and feel confident enough to remain in the generalist setting. And finally they themselves need to be effective life-long learners who can access, critique and apply evidence to questions that constantly arise from clinical practice.

### Discussion in relation to the literature

If one compares these principles to those of McWhinney [21], from a typical developed country context, there are both similarities and differences. Despite the difficult circumstances, the results showed a surprisingly high level of emphasis on patient-centredness and a holistic understanding of the patient. This affirms that a focus on communication skills, a bio-psycho-social clinical model and the doctor-patient relationship is as important a part of training and practice in an African context, as it is in North America and Europe. The need for skills in life-long learning was driven by the clinical challenges that generalists had to constantly deal with in the absence of support from other specialists.

Generalists in Africa did not emphasize the need for them to personally perform home visits, to live in the same environment as their patients (although this was often the case), to think about their patients as a population at risk or to look for opportunities for health promotion and disease prevention in each consultation. Although most of the generalists agreed that a community-orientated approach to primary care was desirable, none of those interviewed were actually engaged in activities that made this practically visible. This paradox was also identified by the linked Delphi study [12] and raises the question as to whether generalists should try harder to implement this principle or look for alternatives. Possible reasons for the failure to implement this principle include a lack of time, role models and skills as well as a lack of priority given to this dimension by the health care system in its expectations, monitoring and evaluation. Health care systems often measure and reward the quantity of care, for example headcounts, rather than the quality of care or impact on health outcomes.

Generalists in Africa articulated a number of different principles such as the need to be competent in a broader range of disciplines (e.g. anaesthetics and surgery) and procedures (e.g. Caesarian sections) at the hospital level in addition to primary care. This is one of the most obvious differences compared to the practice of their northern colleagues, but appears to be limited to those practicing in rural areas. Their role as a manager also went further than just responsibility for the resources utilized in the consultation to a broader responsibility for clinical co-ordination and governance in the facility. This role was always present and yet also held in tension with the pressure of patient care. Their role as a trainer and mentor was also emphasized and driven by their own need to retain and equip a team of health workers to share the demands of patient care.

If the findings are compared with the profile of the 'five-star' family doctor promoted by WONCA and the WHO [22] there is real-life synergy with the roles of care-provider, decision-maker, communicator and manager, but a lack of substance to the role of community leader. Even within these roles some aspects are poorly developed such as continuity of care and health promotion at a community level. From our findings however one could add a sixth role, namely that of clinical trainer and mentor.

Part of the rationale for this study was to see if the principles derived from a panel of local experts in the linked Delphi study [12] were congruent with the principles derived inductively from generalist doctors who were untrained in family medicine but working in typical settings for at least 5 years. Overall the findings are very similar and this helps to confirm the validity of the picture emerging from these studies.

If the principles derived from these generalists are compared with the core dimensions of effective primary care [23] one can see that generalists are particularly focused on issues to do with the comprehensiveness of care and the range of diagnoses, procedures and services that are required for such a comprehensive scope of practice. Beyond this they recognize their role in terms of co-ordination of care within the facility and the need for teamwork as well as appropriate substitution of primary care providers such as clinical nurse practitioners and mid-level health workers as the first contact. They are concerned with the quality and efficiency of care as part of their managerial role and also for improving continuity of care. Continuity is mostly conceived in terms of improving information and management continuity, with the hope of more relational continuity in the future [24]. Access to care was not mentioned and this is probably because the workload is already experienced as high and there is little community-orientated thinking. Likewise equity was not specifically mentioned as an active issue.

### Strengths and limitations of the study

The findings are derived from a group of relatively long-term generalists working at the 'coalface' and speaking from countries in east, west and southern Africa. It was difficult to find generalists with 10 years of experience as originally planned so those with at least 5 years were selected. The interviews were analysed and interpreted in a participatory and rigorous process involving all four authors. The total number of interviews was less than planned and for logistic reasons were conducted by a number of different interviewers. French and Portuguese speaking countries were not represented. It is likely that respondents presented their personal attributes in the best possible light and their colleagues may have given a different picture.

### Implications of the study

Postgraduate training in family medicine should pay attention to the picture of generalist work derived from this and the related Delphi study. Curricula should ensure that clinical training is sufficiently comprehensive to ensure competency across a broad range of diagnoses and procedures. Curricula should also pay more attention to the relational, teaching and organizational skills required for the roles of care provider, manager, trainer and mentor. Curricula should continue to emphasise the knowledge, attitudes and skills required for person-centred and holistic biopsychosocial care. It appears that generalists are currently balancing these roles and there is an assumption that training can better prepare people. It is also clear that the context of training is less than ideal in many settings.

Further study is needed to explore how or even if the concept of community-orientated care can be adopted by the generalist doctor. If so, what is their role or contribution to the realization of this goal? How can the tension between individual patient care within the hospital and clinic and the need to think about the population at risk in a defined district be resolved?

## Conclusion

This study has derived a set of principles for the practice of generalist doctors in sub-Saharan Africa based on their reported activities and thinking. These principles resonate with those derived from an expert panel of family physicians in a linked study. The social, economic, cultural and health system contexts in which generalist doctors function are significantly different to those in the north, resulting in fewer opportunities to refer and a higher level of hospital-based and procedural care in Africa. Although the principles identified in this study differ in some aspects from those articulated for generalist doctors in more developed country settings and by WONCA and the WHO, the core principles of a biopsychosocial approach to patient-centred practice remain common to all approaches. These principles have implications for the postgraduate training of family physicians in sub-Saharan Africa and highlight questions regarding the realization of community-orientated primary care.

## Competing interests

The authors declare that they have no competing interests.

## Authors' contributions

The four authors jointly conceptualized the study. SR led the research team and managed the project, and all the authors participated equally in data collection, analysis and write-up. All the authors read and approved the final manuscript.

## Authors' information

SJR is a family physician, and professor and chair of Primary Health Care in the Faculty of Health Science at the University of Cape Town.

RM is a family physician, and professor and head of the Division of Family Medicine and Primary Care in the Faculty of Health Sciences at the University of Stellenbosch, South Africa.

RD is a family physician, and head of the Department of Family Medicine in the Faculty of Medicine at Moi University, Eldoret, Kenya.

SM is a family physician working in the Johannesburg Metro Department of Family Medicine & Primary Health Care, South Africa

## Pre-publication history

The pre-publication history for this paper can be accessed here:

http://www.biomedcentral.com/1471-2296/12/67/prepub
